# Two Ducts, One Drug—Pembrolizumab-Induced Pancreatic Injury Manifesting as Double-Duct Sign

**DOI:** 10.14309/crj.0000000000001878

**Published:** 2025-11-06

**Authors:** Tareq Alsaleh, Mohamad Khaled Almujarkesh, Abdullah Abbasi, Mustafa Arain, John George

**Affiliations:** 1 Department of Internal Medicine, AdventHealth Orlando, Orlando, FL; 2 Department of Gastroenterology and Hepatology, AdventHealth Orlando, Orlando, FL; 3 Center for Interventional Endoscopy, AdventHealth Orlando, Orlando, FL; 4 Pancreas Center, AdventHealth Orlando, Orlando, FL

**Keywords:** immune checkpoint inhibitors, pancreatitis, pancreas, ERCP, stricture

## Abstract

Immune checkpoint inhibitors (ICIs) can rarely result in ICI-induced pancreatic injury (ICI-PI). Its clinical and radiologic presentation is heterogenous, ranging from asymptomatic radiological signs to severe pancreatitis. Management is mainly with supportive measures and drug discontinuation. In this study, we present an unusual case of ICI-PI, secondary to pembrolizumab, resulting in dilation of both pancreatic and common bile ducts, displaying a “double-duct” sign on imaging. This radiologic sign, commonly associated with malignancy, has not been previously reported with ICI-PI. Endoscopic retrograde cholangiopancreatography with plastic stent placement resulted in quick improvement, highlighting the potential role of endotherapy in treatment of ICI-PI.

## INTRODUCTION

Immune checkpoint inhibitors (ICIs) are used in multiple solid and hematologic malignancies. A rare immune-related adverse effect of these agents is pancreatic injury.^1^ ICI-induced pancreatic injury (ICI-PI) usually presents as incidentally elevated lipase levels with absent-to-mild symptoms or imaging findings.^2^ Management is mainly supportive, with drug discontinuation. In this study, we present a rare case of ICI-PI presenting as symptomatic strictures in both the pancreatic duct (PD) and common bile duct (CBD), a radiological sign more commonly associated with pancreatic malignancy, successfully treated with endoscopic intervention.

## CASE REPORT

Our patient is a 72-year-old woman with a medical history of endometrial adenocarcinoma treated 9 years ago with total abdominal hysterectomy, bilateral salpingo-oophorectomy, and adjuvant chemotherapy, and stage 3a renal cell carcinoma diagnosed a year ago and managed with left radical nephrectomy, followed by adjuvant pembrolizumab at 200 mg every 3 weeks. The patient had tolerated the treatment well with minimal side effects and normal surveillance renal, liver, and thyroid function tests.

On a follow-up visit with her oncologist after the 17th and last cycle of pembrolizumab, the patient reported progressive upper quadrant and epigastric pain associated with nausea and vomiting, which had started gradually over the preceding week. She denied any fevers, chills, diarrhea, or other symptoms. She was vitally stable and not febrile. Physical examination was significant for epigastric tenderness, but there was no jaundice. Her laboratory tests showed an elevated lipase of 354 U/L and a mildly elevated alkaline phosphatase at 109 U/L. Her white blood cell count was normal at 7.46 (10*3/μL). Bilirubin (0.4 mg/dL), aspartate aminotransferase (12 U/L), and alanine aminotransferase (13 U/L) were also unremarkable.

**Figure 1.**
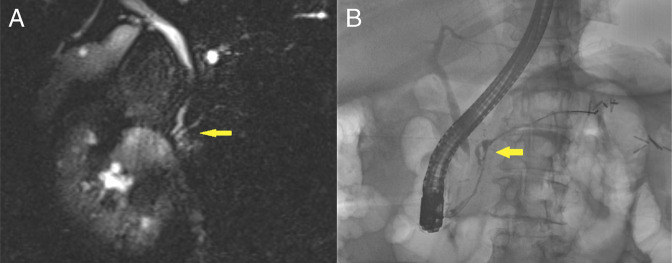
(A) Borderline dilatation in the pancreatic duct and a 9 mm common bile duct, both abruptly terminating in the pancreatic head (yellow arrow), consistent with “double-duct sign.” (B) Cholangiogram showed an indeterminate distal bile duct stricture, and pancreatogram showed a stricture in the neck of the pancreas with mild upstream dilation (yellow arrow).

A surveillance magnetic resonance imaging of the abdomen was performed, which showed a borderline dilatation in the PD and a 9 mm CBD, both of which abruptly terminated in the pancreatic head, which were consistent with “double-duct sign.” There was also mild atrophy of the pancreatic neck, body, and tail without signs of acute pancreatitis (Figure 1).

**Figure 2.**
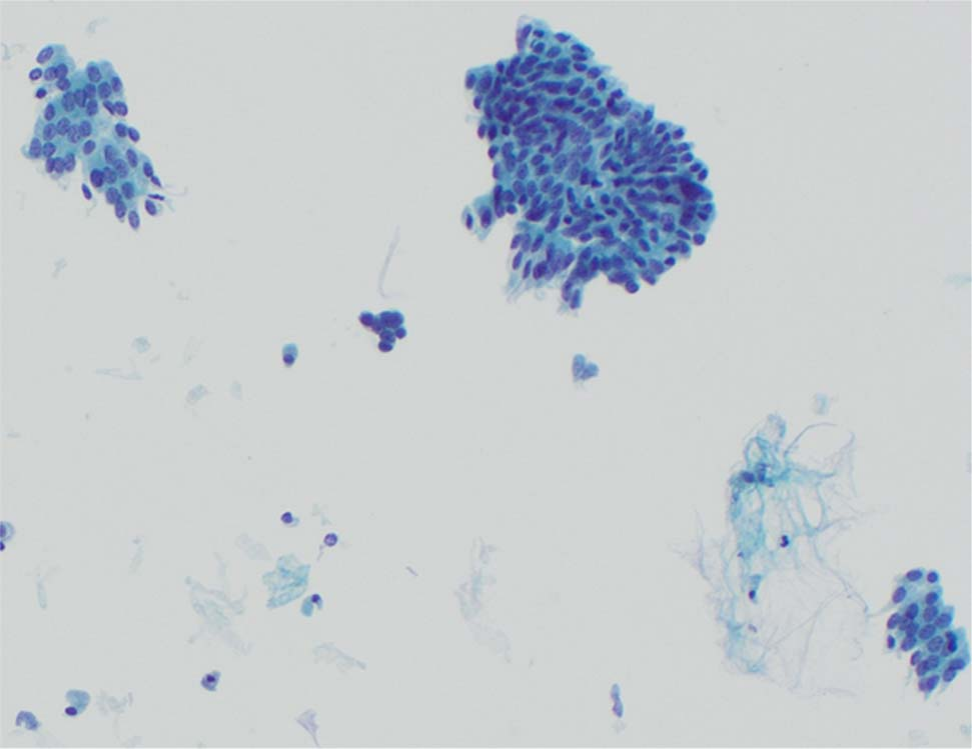
Benign-appearing clusters of glandular epithelial cells on cytology brushings from endoscopic retrograde cholangiopancreatography.

Endoscopic ultrasound (EUS) with endoscopic retrograde cholangiopancreatography (ERCP) were subsequently performed for further evaluation. Under EUS, no discrete mass lesions were seen in the pancreas. There was a PD stricture in the head of the pancreas with mild upstream duct dilation in the pancreatic neck. There was also a stricture in the intrapancreatic portion of the CBD, with mild upstream dilation. The pancreatic parenchyma was atrophic in the body and tail of the pancreas. ERCP showed similar findings to EUS (Figure 1). Cells for cytology were obtained by brushings in the lower third of the CBD. A 5Fr by 9 cm plastic stent was placed in the ventral PD, and a 10Fr by 7 cm plastic stent was placed in the CBD. The patient tolerated the procedure well, with no postoperative complications. Cytology revealed benign-appearing clusters of glandular epithelial cells, with no malignant cells (Figure 2).

The patient denied any history of alcohol drinking or smoking or family history of pancreatic or autoimmune disease. Extensive workup to evaluate other potential causes of these findings in the pancreas was negative. This led to the diagnosis of ICI-PI, and the patient's pembrolizumab was discontinued. Her symptoms dramatically improved following endotherapy, and the decision was made to not start steroids.

**Figure 3.**
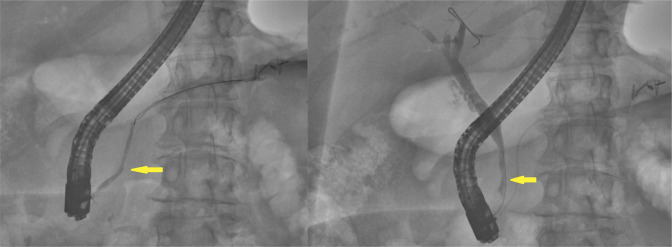
Resolution of the previously seen strictures in the distal bile duct and pancreatic duct (yellow arrows) on follow-up endoscopic retrograde cholangiopancreatography.

Two months later, a repeat ERCP for stent removal showed resolution of the previously seen strictures in the distal bile duct and PD (Figure 3).

## DISCUSSION

Pembrolizumab is an ICI that disinhibits T cells by targeting the regulatory immune checkpoint PD-1 (programmed cell death protein 1). This facilitates a heightened inflammatory response of the immune system that has antitumor effects and immune-related adverse events due to inflammatory damage to multiple organs.^3^ One such immune-related adverse event is ICI-induced pancreatic injury, also known as type 3 autoimmune pancreatitis (AIP). This entity is distinct from chronic pancreatitis and classic AIP due to its largely asymptomatic presentation.^4^ ICI-PI is more common with ICIs targeting CTLA-4 (Cytotoxic T-lymphocyte-associated protein 4) compared with those targeting PD-1, and the risk is highest in combination therapy.^5^

The proposed immunological mechanisms underlying ICI-PI involves neutrophil and CD8^+^ T-cell-predominant infiltrates, which induce acinar cell atrophy and acinar-ductal metaplasia while preserving the pancreas' lobular architecture.^6,7^ In a report by Rawson et al, neutrophilic infiltrates were also observed in the pancreatic ducts.^8^ Moreover, ICI-induced cholangitis has been associated with periductal lymphocytic infiltration and fibrosis.^9^ These findings highlight the potential for ICIs to simultaneously affect the PD and CBD as seen with our patient.

ICI-PI has a wide spectrum of clinical presentations, including asymptomatic elevations of pancreatic enzymes, incidental imaging findings of pancreatitis, or symptomatic painful pancreatitis. The most common symptoms are epigastric pain (39%), nausea and vomiting (28%), and fever (9%), but most patients have an asymptomatic presentation. Imaging can reveal findings of acute pancreatitis, such as segmental hypoenhancement and peripancreatic fat stranding.^1^ Signs suggesting chronic injury can be seen as well. These include pancreatic volume loss and increasing pancreas/spleen attenuation ratio.^4^ This is consistent with our patient's presentation, with atrophic parenchymal changes on imaging and EUS.

However, the radiological appearance of the double-duct sign on magnetic resonance imaging, the PD and CBD dilation on EUS, and benign cytology findings on fine-needle aspiration (FNA) are unusual for ICI-PI. The double-duct sign describes the combined dilatation of the CBD and PD, often caused by pancreatic malignancy.^10^ It may also be due to benign causes, such as chronic pancreatitis, choledocholithiasis, pancreatic cysts, anatomical variations, and common channel fibrotic strictures. The absence of jaundice makes these benign etiologies more likely. However, there are no reported cases due to ICI-PI use.^11,12^

ICI-PI is diagnosed when there is a temporal pattern of ICI initiation before symptom onset, and other potential causes are ruled out. These include alcohol consumption, hypertriglyceridemia, biliary pathology, AIP, pancreatic cystic lesions, and neoplastic processes.^13^

The mainstay of management of ICI-PI is aggressive intravenous fluid hydration, pain control, and stopping ICI therapy.^14^ In moderate pancreatic injury, a 4–6-week steroid taper may be used, with possible resumption of ICIs eventually. In severe cases, they should be permanently discontinued.^15^ However, there was no observed benefit of steroids in preventing long-term adverse outcomes of ICI-PI or improving overall survival.^1^ Our case demonstrates the ability to delineate ductal anatomy and obtain samples for cytopathologic evaluation with endoscopy, allowing thorough evaluation while avoiding invasive surgeries. Moreover, our patient's clinical response without the use of steroids or immunosuppressive medication highlights its therapeutic potential in this structuring phenotype.

Although double-duct sign is commonly associated with pancreatic head cancer, our case underscores the importance of considering ICI-PI as a possible culprit. Evaluating other potential causes is paramount, and treatment is supportive with holding the ICI or discontinuing it in severe cases. Further research is needed to define the role of steroids and immunosuppressants in management, as our patient's response without using steroids further suggests that immunosuppression is not required in all symptomatic cases and highlights the important role of endoscopic interventions.

## DISCLOSURES

Author contributions: T Alsaleh, and MK Almujarkesh conducted literature reviews, gathered the necessary patient information, and wrote/edited the paper. A. Abbasi conceived the project and assisted with writing/editing. M. Arain and J. George supervised the project and provided guidance. J. George is the article guarantor.

Financial disclosure: All expenses related to this case report were solely funded by the AdventHealth Orlando Department of Graduate Medical Education.

Informed consent was obtained for this case report.

## ABBREVIATIONS:

AIP: autoimmune pancreatitis
CBD: common bile duct
CTLA-4: Cytotoxic T-lymphocyte-associated protein 4
ERCP: endoscopic retrograde cholangiopancreatography
EUS: endoscopic ultrasound
ICI: immune checkpoint inhibitors
ICI-PI: immune checkpoint inhibitor-induced pancreatic injury
PD: pancreatic duct
PD-1: programmed cell death protein 1
